# Serum VEGF and ANGPT1 as angiogenesis markers may predict the outcomes of older adults with hip fractures

**DOI:** 10.3389/fmed.2025.1654448

**Published:** 2025-10-21

**Authors:** Yuan Yao, Yachang Xing, Zhibang Zhao, Wenliang Fan, Qingbo Chu

**Affiliations:** Emergency Trauma Center, Nanyang Second People’s Hospital, Nanyang, Henan, China

**Keywords:** VEGF, ANGPT1, ANGPT2, hip fracture, outcome

## Abstract

**Objective:**

This study aims to investigate the potential of serum Vascular Endothelial Growth Factor (VEGF) and Angiopoietin 1 (ANGPT1) as angiogenesis markers to predict the outcomes of older adults with hip fractures.

**Methods:**

An observational study was conducted at the Emergency Trauma Center of Nanyang Second People’s Hospital. Serum VEGF and ANGPT1 were measured on the first morning after surgery. Patients were followed up for 1 year to assess survival status and the ability to walk freely at 3, 6, and 12 months post-surgery. Receiver operating characteristic (ROC) curves were constructed to determine the predictive power of these markers, and propensity score matching (PSM) was performed to account for confounding factors. Multivariate Cox regression and logistic regression models were used to further analyze the prognostic roles of these markers.

**Results:**

The study cohort included 380 patients, with a mean age of 75.71 ± 8.58 years and a mortality rate of 17.11% within 1 year. Kaplan–Meier survival analysis revealed that low levels of VEGF and ANGPT1 were significantly associated with decreased survival probability. Multivariate Cox regression models indicated that low VEGF and ANGPT1 were independent risk factors for one-year mortality, while ANGPT2 did not show significant prognostic value.

**Conclusion:**

Elevated serum levels of VEGF and ANGPT1 are associated with improved outcomes in older adults with hip fractures, highlighting the importance of angiogenesis in fracture healing.

## Introduction

Hip fractures are a major health concern among older adults (1). These fractures are primarily caused by falls or low-impact trauma and are often associated with age-related bone density reduction, osteoporosis, and underlying health conditions (2). The mortality rate within 1 year following a hip fracture or surgery ranges from 17 to 25% (2, 3). Most of these deaths are due to complications during fracture healing, such as pneumonia, pressure ulcers, and thromboembolism (4). Identifying risk factors for hip fracture prognosis is crucial for improving patient outcomes and reducing mortality rates (5). This is not only important for guiding clinical interventions and resource allocation, but also for developing effective prevention and treatment strategies.

Bone healing is a complex and dynamic process that involves multiple stages, including inflammation, repair, and remodeling (6). In the case of hip fractures, the process of bone healing is particularly critical due to the weight-bearing nature of the hip joint and the potential for complications such as nonunion or delayed union (7). The inflammatory phase initiates the healing cascade, with the formation of a hematoma and the recruitment of inflammatory cells to the site of injury (8). This is followed by the repair phase, during which granulation tissue forms and osteoblasts begin to produce new bone matrix. Finally, the remodeling phase continues for months to years, during which woven bone is converted into lamellar bone. Angiogenesis plays a vital role in this process by providing necessary nutrients and oxygen to the healing site and removing metabolic waste, thereby promoting the proliferation and differentiation of osteoblasts and the formation of new bone tissue (9, 10). However, if the process of fracture healing is disrupted, it may lead to various complications, which in turn affect the functional recovery and quality of life of patients.

Previous studies have found that angiogenesis-related markers are elevated following hip fractures, which may be a compensatory response of the body (11). Individual differences in angiogenesis markers may have the potential to predict the prognosis of hip fractures. In this study, we selected Vascular Endothelial Growth Factor (VEGF), Angiopoietin 1 (ANGPT1), and Angiopoietin 2 (ANGPT2) as angiogenesis markers and followed up on elderly patients after hip fracture surgery to verify their predictive power for outcomes.

VEGF is rapidly up-regulated by hypoxia-inducible factors in the fracture hematoma, where it triggers endothelial proliferation and directs capillary ingrowth. ANGPT1, secreted by peri-vascular cells, subsequently binds Tie-2 receptors to stabilize nascent vessels, reduce permeability, and prevent endothelial apoptosis (12). This VEGF–ANGPT1 sequence converts fragile capillary sprouts into a mature, perfused network that delivers oxygen and anabolic factors required for osteoblast differentiation and mineral deposition (13). Disruption of either signal impairs neovascularization and is associated with delayed union, non-union, and increased post-operative mortality (14). By quantifying these specific ligands, we therefore aimed to capture the functional integrity of the angiogenic response and test whether circulating levels forecast long-term survival and mobility after hip fracture.

We hypothesize that these markers may serve as valuable indicators for assessing the risk of adverse outcomes in hip fracture patients. By understanding the role of angiogenesis in fracture healing and the potential predictive value of these markers, we aim to provide a scientific basis for early intervention and improved patient management. This research not only contributes to the understanding of the biological mechanisms underlying fracture healing but also offers a practical approach to enhance the prognosis of hip fracture patients.

## Methods

### Study design

**Figure 1:**
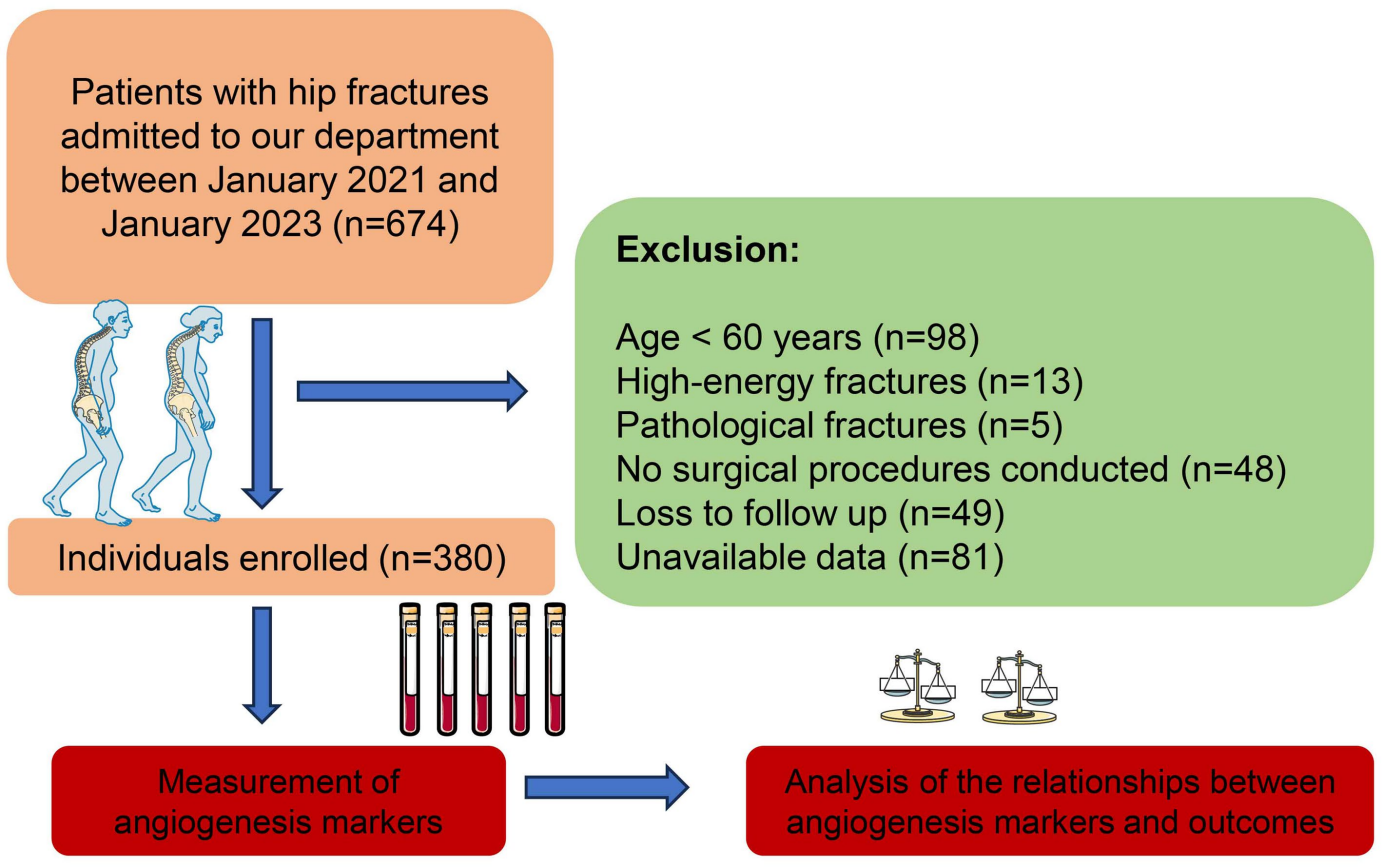
Flowchart of our study.

This study was carried out as an observational investigation at the Emergency Trauma Center of Nanyang Second People’s Hospital, Nanyang, Henan Province, China. The research complied with the ethical principles outlined in the Declaration of Helsinki and received approval from the Ethics Committee of Nanyang Second People’s Hospital (ID: 2020 Research Review No. 13). The study cohort consisted of older patients with hip fractures admitted to our department from January 2021 to January 2023. Eligibility for inclusion required patients to meet the following criteria: a. aged 60 years or older; b. low-energy fractures; c. provision of informed consent. Patients were excluded if they had: a. pathological fractures; b. no surgical procedures conducted; c. loss to follow up; d. unavailable data. After applying the inclusion and exclusion criteria, the final study group was established (Figure 1). Robust protocols were put in place to ensure patient confidentiality, and all participants provided explicit written consent prior to their involvement in the study.

### Baseline data

The baseline characteristics of participants were obtained from our hospital’s electronic medical records. These included age, sex, BMI, fracture history, and smoking and alcohol use history, as well as comorbidities, electrocardiogram findings, and chest X-ray results. The Charlson comorbidity index (CCI) was calculated to assess the impact of comorbid conditions (15). Upon admission, RBC, Hb, GLU, and ALB levels were measured in the laboratory department using routine hospital equipment (Sysmex XE-2100, Kehua Bio-engineering Co., Ltd., Shanghai, China; TBA-120FR, Toshiba Co., Ltd., Tokyo, Japan), with data recorded in the electronic medical records.

### Elisa

Serum VEGF, ANGPT1, and ANGPT2 were measured on the first morning after the surgery using a human VEGF ELISA kit (PV963, Beyotime), ANGPT1 ELISA kit (JL10166-96 T, Jonlnbio), and ANGPT2 ELISA kit (JL10504-96 T, Jonlnbio) following the manufacturer’s protocols. Briefly, blood samples from participants were collected and processed following the kit’s instructions to ensure they were free from contaminants that could interfere with the assay. The samples were added to antibody-coated plates. After incubation and washing, bound VEGF, ANGPT1, and ANGPT2 were detected using HRP-conjugated antibodies and a colorimetric substrate. The absorbance was measured, and the concentrations of these markers were determined from a standard curve constructed with known concentrations of VEGF, ANGPT1, and ANGPT2. Laboratory staff performing the assays were blinded to all clinical outcomes and survival data.

### Follow-up and outcomes

Patients were followed up for a duration of 1 year. For those who regularly attended our outpatient clinic, their health status was recorded by our medical staff. As for other patients, we conducted follow-up via telephone. In our study, survival time was defined as the interval from the date of surgery to the date of death due to any disease. Patients who survived for over 1 year were categorized as censored data. Patients who could independently perform daily activities without assistance were considered to have the ability to walk freely. The outcomes under investigation in this study included survival status and the ability to walk freely at 3 months, 6 months, and 1 year post-surgery.

### Statistical analyses

Continuous variables are expressed as mean ± standard deviation, and categorical variables as counts with percentages. For continuous variables, data following a normal distribution were analyzed using independent Student’s t-tests, while non-normally distributed data were assessed with Wilcoxon rank-sum tests. Categorical variables were examined using Chi-squared tests or Fisher’s exact test, as appropriate.

Baseline characteristics of patients grouped by one-year survival status were summarized and compared. Then, a 1:1 propensity score matching (PSM) with a caliper of 0.2 was performed to reduce the influence of confounding factors. Post-matching baseline features were also re-examined to ensure comparability between groups. Receiver operating characteristic (ROC) curves were established to assess the role of each marker in outcomes of hip fractures and identify the optimal cutoff values based on the Youden index. Patients were grouped into normal and high marker levels according to these cutoffs, and outcomes were compared between these groups. Cox and Logistic regression models were constructed to further elucidate the predictive roles of VEGF, ANGPT1, and ANGPT2 while accounting for co-variables. A *p* value of less than 0.05 was considered significant. All statistical analyses were conducted using R software version 4.2.2 (R Foundation for Statistical Computing, Vienna, Austria).

## Results

### General characteristics

Ultimately, 380 patients were enrolled, of whom 65 died within 1 year. The baseline characteristics of populations before and after PSM were summarized in Table 1 and Supplementary Table 1. Among all the patients, 250 (65.79%) were female and 130 (34.21%) were male; the average age was 75.71 ± 8.58 years, with a BMI of 21.91 ± 4.31. Two hundred and five patients (53.95%) had femoral neck fractures, while 175 patients (46.05%) had intertrochanteric fractures. When comparing the baseline characteristics between patients who died within 1 year and survivors, significant differences were found in age, sex, and GLU levels (Table 1), prompting the use of propensity score matching (PSM). After PSM, 124 patients (62 pairs) were included, and no significant differences were observed in their baseline characteristics (Supplementary Table 1).

**Table 1:** Baseline characteristics of populations included in our study.

Variables	Unmatched populations (*n* = 380)			*p*-value
	Overall	Survival > 1 year	Survival ≤ 1 year	
	(*n* = 380)	(*n* = 315)	(*n* = 65)	
Age (years)	75.71 ± 8.58	75.21 ± 8.64	78.11 ± 7.88	0.011
BMI (kg/m^2^)	21.91 ± 4.31	21.95 ± 4.39	21.68 ± 3.92	0.678
Sex (female)	250 (65.79%)	217 (68.89%)	33 (50.77%)	0.005
Fractures history (yes)	46 (12.11%)	37 (11.75%)	9 (13.85%)	0.636
Smoking history (yes)	39 (10.26%)	34 (10.79%)	5 (7.69%)	0.453
Alcoholism history (yes)	24 (6.32%)	17 (5.40%)	7 (10.77%)	0.18
Location of fracture (femoral neck)	205 (53.95%)	164 (52.06%)	41 (63.08%)	0.105
Surgical procedures (arthroplasty)	189 (49.74%)	151 (47.94%)	38 (58.46%)	0.122
Anesthesia (spinal)	4 (1.05%)	4 (1.27%)	0 (0.00%)	> 0.999
CCI score (>4)	90 (23.68%)	73 (23.17%)	17 (26.15%)	0.607
Electrocardiogram (abnormal)	216 (56.84%)	180 (57.14%)	36 (55.38%)	0.794
Chest radiograph (abnormal)	191 (50.26%)	159 (50.48%)	32 (49.23%)	0.855
Hypertension (yes)	225 (59.21%)	191 (60.63%)	34 (52.31%)	0.214
Polytrauma (yes)	55 ± 14.47	42 ± 13.33	13 ± 20.00	0.164
Time from injury to surgery (Days)	4.89 ± 0.94	4.88 ± 0.93	4.92 ± 0.97	0.502
RBC (10^12/L)	4.63 ± 0.71	4.65 ± 0.70	4.53 ± 0.78	0.315
Hb (g/L)	96.93 ± 15.07	96.64 ± 14.85	98.35 ± 16.09	0.371
ALB (g/L)	38.08 ± 8.78	38.04 ± 8.78	38.27 ± 8.86	0.887
GLU (mmol/L)	6.36 ± 1.43	6.43 ± 1.41	6.03 ± 1.49	0.038
VEGF (pg/mL)	159.36 ± 55.25	162.20 ± 56.85	145.58 ± 44.56	0.027
ANGPT1 (ng/mL)	31.01 ± 12.84	32.61 ± 12.43	23.25 ± 12.03	<0.001
ANGPT2 (ng/mL)	3.35 ± 1.05	3.38 ± 1.10	3.19 ± 0.78	0.366

Continuous variables were expressed as mean ± standard deviation and categorical variables were presented as count (percent). BMI, body mass index; CCI, Charlson comorbidity index; Hb, Hemoglobin; RBC, red blood count; GLU, blood glucose; ALB, albumin; VEGF, vascular endothelial growth factor; ANGPT1, angiopoietin 1; ANGPT2, angiopoietin 2.

### Predictive abilities of angiogenesis markers

**Figure 2:**
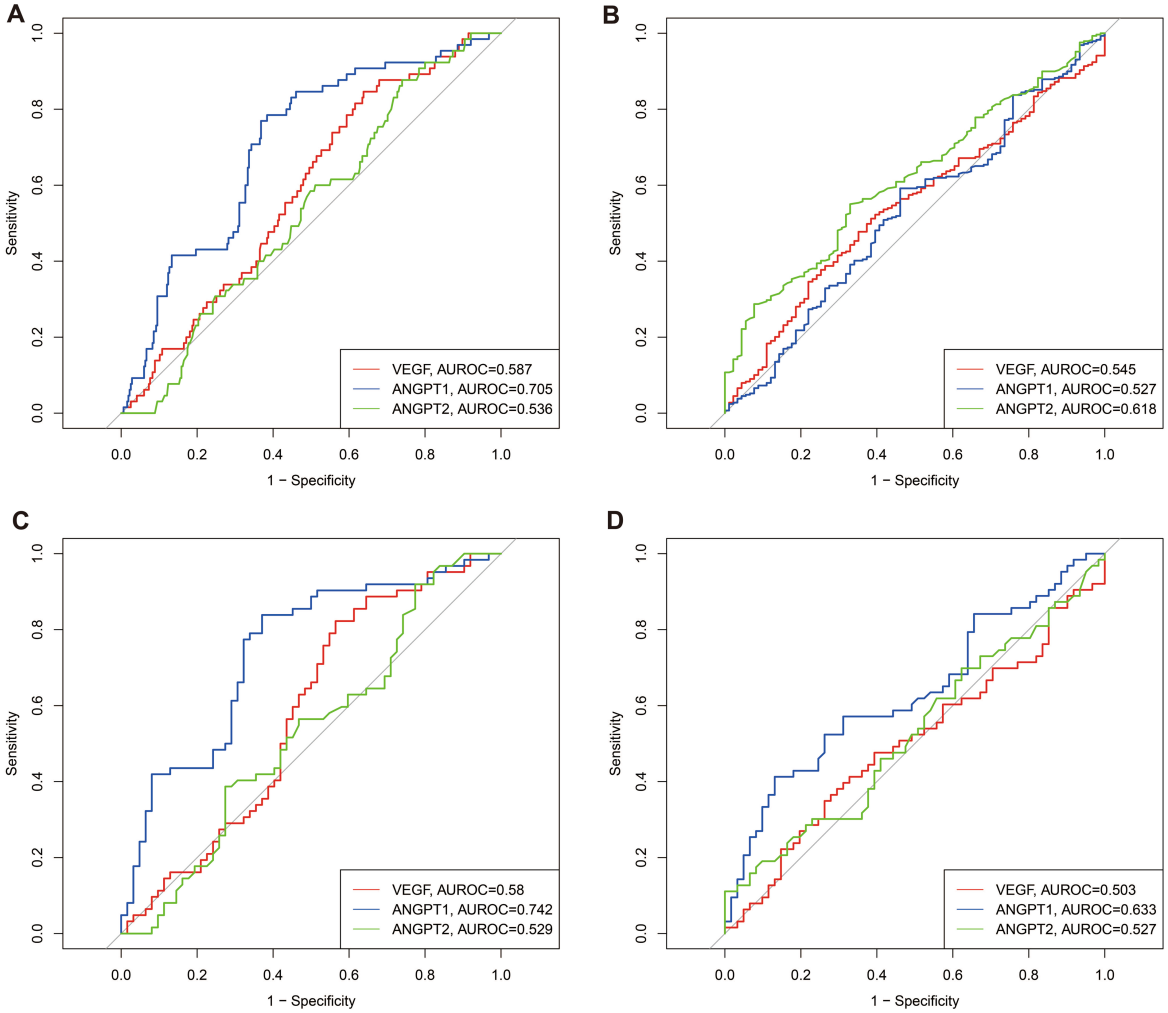
Predictive abilities of VEGF, ANGPT1, and ANGPT2 for 1-year survival and 1-year free walking abilities in unmatched and matched populations. **(A)** for -year survival in unmatched populations; **(B)** for -year free walking abilities in unmatched populations; **(C)** for -year survival in matched populations; **(D)** for -year free walking abilities in matched populations.

To further elucidate the predictive capacity of angiogenesis markers, we constructed ROC curves to assess the ability of VEGF, ANGPT1, and ANGPT2 to predict one-year mortality and free walking ability (Figure 2). In the unmatched cohort, the areas under the ROC curve (AUROC) for one-year mortality were 0.587 for VEGF, 0.705 for ANGPT1, and 0.536 for ANGPT2 (Figure 2A). For one-year free walking ability, the AUROC values were 0.587 for VEGF, 0.509 for ANGPT1, and 0.610 for ANGPT2. Similarly, in the matched cohort, the AUROC values for one-year mortality were 0.580 for VEGF, 0.742 for ANGPT1, and 0.529 for ANGPT2, while for free walking ability, the values were 0.535 for VEGF, 0.612 for ANGPT1, and 0.528 for ANGPT2. Based on the ROC curves and Youden index in the matched population, we determined the optimal cutoff values for these markers. VEGF levels below 181.55 pg./mL were classified as low VEGF, ANGPT1 levels below 32.72 ng/mL were classified as low ANGPT1, and ANGPT2 levels below 2.22 ng/mL were classified as low ANGPT2.

### Outcomes

**Figure 3:**
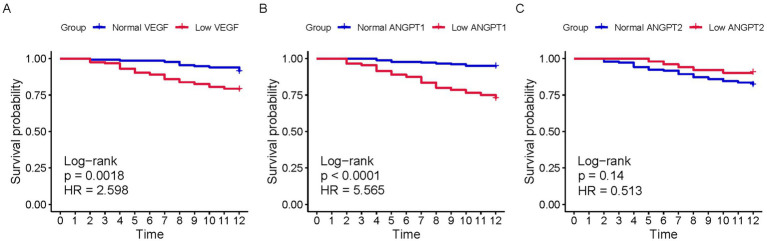
K-M curves of populations with different serum levels of VEGF, ANGPT1, and ANGPT2 for 1-year survival. **(A)** VEGF; **(B)** ANGPT1; **(C)** ANGPT2.

To further investigate the predictive capacity of angiogenesis markers, we divided the population into groups based on the cutoff values of VEGF, ANGPT1, and ANGPT2. Kaplan–Meier (KM) curves were constructed (Figure 3). Patients with low VEGF (log-rank *p* = 0.002) and low ANGPT1 (log-rank *p* < 0.001) showed significantly lower survival probability than the normal group. No significant difference in mortality probability was observed between the two groups divided by ANGPT2. Moreover, the outcomes of patients in different groups were compared. Consistent with the KM curves, low VEGF and ANGPT1 were associated with low 6-month (VEGF *p* = 0.001, ANGPT1 *p* < 0.001; Table 2) and 1-year (VEGF *p* = 0.002, ANGPT1 *p* < 0.001, Table 2) mortality rates, but not with free walking ability. ANGPT2 showed no significant association with any of the outcomes (Supplementary Table 2).

**Table 2:** Outcomes of populations grouped by different angiogenesis markers.

Outcomes	Overall	Normal VEGF	Low VEGF	*p*-value	Normal ANGPT1	Low ANGPT1	*p*-value
	(*n* = 380)	(*n* = 133)	(*n* = 247)		(*n* = 180)	(*n* = 200)	
3-month mortality	9 (2.37%)	1 (0.75%)	8 (3.24%)	0.243	0 (0.00%)	9 (4.50%)	0.011
6-month mortality	29 (7.63%)	2 (1.50%)	27 (10.93%)	0.001	4 (2.22%)	25 (12.50%)	<0.001
1-year mortality	65 (17.11%)	12 (9.02%)	53 (21.46%)	0.002	10 (5.56%)	55 (27.50%)	<0.001
3-month free walking rate	90 (23.68%)	32 (24.06%)	58 (23.48%)	0.899	49 (27.22%)	41 (20.50%)	0.124
6-month free walking rate	219 (57.63%)	69 (51.88%)	150 (60.73%)	0.096	106 (58.89%)	113 (56.50%)	0.638
1-year free walking rate	289 (76.05%)	98 (73.68%)	191 (77.33%)	0.427	143 (79.44%)	146 (73.00%)	0.142

VEGF, vascular endothelial growth factor; ANGPT1, angiopoietin 1; ANGPT2, angiopoietin 2.

### Multivariate analyses

To further reduce bias from confounding variables, we established multivariable models. Initially, we conducted univariate Cox regression (Supplementary Table 3) to examine the relationship between each variable and mortality risk. Variables significant in univariate analysis were included in the multivariable Cox regression. Both continuous and dichotomized forms of VEGF and ANGPT1 were significantly associated with reduced mortality risk in the multivariable model (Table 3), while ANGPT2 showed no significant association. Similarly, univariate and multivariate logistic regression were performed (Table 4; Supplementary Table 4). Consistent with Cox regression results, continuous and dichotomized VEGF and ANGPT1 effectively predicted 6-month and 1-year mortality (Table 3), whereas ANGPT2 did not. Notably, continuous ANGPT2 was a significant predictor of 6-month and 1-year free walking ability in multivariate models, while VEGF and ANGPT1 were not.

**Table 3:** Cox models of different angiogenesis markers for 1-year survival.

Variables	Univariate model		Multivariate model	
	HR (95% CI)	*p*	HR (95% CI)	*p*
VEGF (continuous)	0.995 [0.990, 0.999]	0.021	0.995 [0.990, 0.999]	0.03
ANGPT1 (continuous)	0.950 [0.933, 0.968]	<0.001	0.947 [0.928, 0.965]	<0.001
ANGPT2 (continuous)	0.859 [0.673, 1.096]	0.221	0.862 [0.675, 1.101]	0.235
Low VEGF	2.616 [1.398, 4.896]	0.003	2.646 [1.410, 4.968]	0.002
Low ANGPT1	5.641 [2.875, 11.068]	<0.001	5.756 [2.932, 11.299]	<0.001
Low ANGPT2	0.511 [0.205, 1.271]	0.149	0.511 [0.205, 1.277]	0.151

VEGF, vascular endothelial growth factor; ANGPT1, angiopoietin 1; ANGPT2, angiopoietin 2.

**Table 4:** Logistics models of different angiogenesis markers for 6-month and 1-year survival and free walking ability.

Variables	6-month mortality		1-year mortality		6-month free walking ability		1-year free walking ability	
	OR (95% CI)	*p*	OR (95% CI)	*p*	OR (95% CI)	*p*	OR (95% CI)	*p*
VEGF (continuous)	0.987 [0.979, 0.995]	0.002	0.994 [0.989, 0.999]	0.03	0.997 [0.993, 1.000]	0.073	0.998 [0.994, 1.002]	0.366
ANGPT1 (continuous)	0.945 [0.915, 0.974]	<0.001	0.937 [0.914, 0.959]	<0.001	1.003 [0.987, 1.019]	0.719	1.009 [0.990, 1.028]	0.34
ANGPT2 (continuous)	1.092 [0.753, 1.548]	0.628	0.823 [0.617, 1.080]	0.172	1.311 [1.071, 1.617]	0.01	1.639 [1.262, 2.171]	<0.001
Low VEGF	10.234 [2.870, 66.055]	0.002	2.934 [1.525, 6.065]	0.002	1.434 [0.935, 2.202]	0.098	1.214 [0.733, 1.993]	0.446
Low ANGPT1	6.276 [2.349, 21.815]	0.001	6.980 [3.512, 15.255]	<0.001	0.908 [0.602, 1.367]	0.643	0.711 [0.435, 1.151]	0.168
Low ANGPT2	0.473 [0.074, 1.686]	0.323	0.489 [0.162, 1.202]	0.154	0.818 [0.451, 1.491]	0.507	0.692 [0.364, 1.363]	0.272

VEGF, vascular endothelial growth factor; ANGPT1, angiopoietin 1; ANGPT2, angiopoietin 2.

## Discussion

This study aimed to evaluate the prognostic role of angiogenesis markers, including VEGF, ANGPT1, and ANGPT2, in older adults with hip fractures. Our results showed that elevated levels of VEGF and ANGPT1 were associated with reduced mortality risk, while ANGPT2 did not exhibit significant predictive value. Notably, continuous ANGPT2 levels could predict free walking ability at 6 months and 1 year. These findings highlight the complex roles of angiogenesis markers in fracture healing and functional recovery.

Angiogenesis, the formation of new blood vessels, is intricately linked to bone regeneration (16). During bone healing, angiogenesis supplies the fracture site with essential nutrients and oxygen, facilitating the survival and function of bone-forming cells like osteoblasts (17, 18). These new vessels not only nourish the repair site but also aid in removing debris, supporting the sequential processes of inflammation, bone formation, and remodeling (19). Furthermore, the cross-talk between angiogenesis and osteogenesis is underscored by the presence of shared signaling pathways and cellular interactions (20). Conversely, bone matrix components can influence vascular stability and function (21). This dynamic interplay between angiogenesis and bone regeneration is crucial for the efficient healing of fractures and the restoration of bone integrity. In hip fractures, the formation of new blood vessels is essential for removing debris and supporting bone regeneration (22).

VEGF plays a critical role in bone healing by promoting angiogenesis, which is essential for the repair and regeneration of bone tissue (23). During the inflammatory phase of bone healing, VEGF is concentrated in the fracture hematoma and is induced by hypoxia (16). It facilitates the release of neutrophils from bone marrow into the circulation and their recruitment to the injury site (24). Furthermore, VEGF is involved in the recruitment of macrophages and the stimulation of angiogenesis, which are crucial for the repair process (25). Our study found that VEGF levels were associated with reduced mortality risk, consistent with prior research indicating that adequate VEGF levels are crucial for effective fracture healing. However, the lack of association between VEGF and functional outcomes suggests that while VEGF influences survival, its role in functional recovery may be limited or mediated by other factors.

ANGPT1 was also found to be associated with reduced mortality risk in our study. ANGPT1 is essential for the stabilization of newly formed blood vessels during the bone healing process (26). It interacts with the Tie2 receptor on endothelial cells, promoting cell–cell adhesion, reducing vascular permeability, and increasing the osteogenesis ability (27). This helps to form a stable vascular network that supports nutrient and oxygen delivery to the fracture site (26). ANGPT1 works in concert with other angiogenic factors like VEGF (28). While VEGF drives the initial sprouting of new blood vessels, ANGPT1 helps to organize and limit the angiogenic response, ensuring that the new vessels are functional and integrated into the existing vascular network (14, 29). This balance is crucial for efficient bone repair. “Mechanistically, ANGPT1 constitutively activates Tie-2 on endothelial cells, tightening cell–cell junctions and reducing vascular permeability. During fracture healing this stabilizes the immature neovasculature generated by VEGF, thereby sustaining nutrient delivery at the hematoma–bone interface (26). Because ANGPT1 is released primarily by peri-vascular cells, its circulating level may mirror the global integrity of the revascularization scaffold more closely than VEGF, which is transiently abundant in the inflammatory hematoma (14, 27, 29) This autocrine stabilizing function could explain why ANGPT1 showed superior discriminative performance for long-term survival in our ROC models.

Unlike ANGPT1, continuous or dichotomized ANGPT2 was not associated with survival in either Cox or logistic models. This neutral prognostic effect may reflect the context-dependent, bidirectional activity of ANGPT2. Mechanistically, ANGPT2 is stored in Weibel-Palade bodies of endothelial cells and is rapidly released upon stimulation by hypoxia, inflammatory cytokines, or mechanical stress at the fracture site (30). Once released, ANGPT2 forms oligomers that can either stabilize or destabilize vessels depending on the local VEGF concentration (31). ANGPT2 interacts with the TIE2 receptor and plays a complex role in bone healing (32, 33). At high concentrations, ANGPT2 can both activate and inhibit TIE2 (34, 35). It can induce TEK/TIE2 tyrosine phosphorylation even in the absence of ANGPT1, thereby activating the PI3K p85 subunit and Akt phosphorylation at Ser473 (36–38). This process promotes cell survival and proliferation (39). However, in the absence of angiogenesis inducers like VEGF, ANGPT2 may induce endothelial cell apoptosis and vascular regression by loosening cell-matrix contacts (35, 40–42). When acting synergistically with VEGF, ANGPT2 promotes endothelial cell migration and proliferation, serving as a permissive angiogenic signal and participating in lymphangiogenesis regulation (40, 41). During bone healing, ANGPT2 helps regulate the balance between angiogenesis and vascular regression, influencing the formation and stability of blood vessels at the fracture site (32, 33). This affects nutrient and oxygen supply to healing bone tissue, ultimately impacting bone regeneration.

Our cohort reflects the standardized peri-operative pathway implemented at Nanyang Second People’s Hospital, which may explain the lower mean BMI (21.9 kg m^−2^) and 17% one-year mortality compared with many Western registries. All patients underwent surgery within 48 h of admission (median 24 h), received spinal or combined anesthesia, and were mobilized by a physiotherapist on the first post-operative day. Early discharge (median length of stay 5 days) to community hospitals or home with ongoing nurse-led care is routine, and total arthroplasty is preferred for displaced femoral-neck fractures. These factors—together with lower prevalence of severe obesity and different discharge destinations—may attenuate post-operative complications and mortality relative to systems with longer acute-care stays. While this enhances internal validity, it also limits generalizability; validation in centers with alternative surgical techniques, delayed mobilization, or higher-intensity inpatient rehabilitation is therefore warranted.

Beyond prognostication, post-operative VEGF and ANGPT1 levels could be integrated into existing orthogeriatric care pathways to personalize management. Patients with low concentrations might be prioritized for enhanced nutritional support, tighter anemia correction, or early referral to aggressive rehabilitation protocols, while those with markedly depressed ANGPT1 could be considered for adjunctive pro-angiogenic strategies. A simple blood sample taken on the first post-operative morning could therefore serve as a rapid, low-cost triage tool to flag high-risk individuals before complications become clinically evident, enabling resource allocation and follow-up intensity to be tailored to biological, rather than purely chronological, risk.

Our study has several limitations. First, as an observational study, we cannot establish causality between angiogenesis markers and patient outcomes. Second, markers were quantified only once (on the first post-operative morning), which does not reflect the dynamic fluctuations of angiogenesis during the inflammatory, reparative, and remodeling phases. Serial measurements of VEGF, ANGPT1, and ANGPT2 will be incorporated into our ongoing multicenter protocol to model individual angiogenic trajectories and their association with hip fractures. Third, our study focused on short-term outcomes (up to 1 year), and the long-term prognostic value of these markers remains to be determined. We have therefore extended the follow-up phase of our future project to 5 years, with scheduled clinical, radiographic, and functional evaluations at 12, 24, 36, and 60 months to determine the long-term prognostic value of VEGF and ANGPT1 for implant survival and joint function. Fourth, our propensity-score analysis was constrained to 1:1 matching because 1:2 or 1:3 algorithms within a 0.2-SD caliper left too few controls in the common-support region; this choice reduced residual confounding but at the price of diminished sample size and statistical power. Additionally, functional outcome was recorded as a binary ‘free walking’ variable, which does not capture the spectrum of disability; standardized instruments such as the Barthel Index or Harris Hip Score would provide a more comprehensive assessment. In our future protocol, we would replace the binary endpoint with the Harris Hip Score and Barthel Index, enabling detailed evaluation of pain, mobility, and activities of daily living. Finally, the single-center recruitment from Nanyang Second People’s Hospital may limit generalizability to other ethnic, nutritional, or health-care settings. Future multicenter studies involving different geographic regions and racial groups are required to validate the prognostic thresholds we report.

In conclusion, our study provides evidence that VEGF and ANGPT1 may serve as prognostic markers for mortality in older adults with hip fractures, while ANGPT2 may have a role in predicting functional recovery. These findings underscore the importance of angiogenesis in fracture healing and suggest potential targets for therapeutic intervention. Future research should explore the dynamic changes in angiogenesis markers during fracture healing and their long-term prognostic significance.

## Data Availability

The raw data supporting the conclusions of this article will be made available by the authors, without undue reservation.
